# Disulfide‐Induced Inhibition of Epoxy Cationic Photopolymerization: A Route to Maskless Patterning

**DOI:** 10.1002/marc.202500956

**Published:** 2026-02-05

**Authors:** Alberto Spessa, Roberta Bongiovanni, Alessandra Vitale

**Affiliations:** ^1^ Department of Applied Science and Technology Politecnico Di Torino Torino Italy; ^2^ INSTM‐Politecnico Di Torino Research Unit Firenze Italy

**Keywords:** disulfides, epoxides, maskless photolithography, patterning, ring‐opening polymerization, UV‐curable coatings

## Abstract

In photoinduced cationic ring‐opening polymerization (ROP) of epoxy monomers, chain‐transfer reactions in the presence of alcohols are well established, and monosulfides are known to inhibit polymerization even at very low loadings (i.e., 0.01 mol%). Herein, it is thoroughly investigated the role of a disulfide‐containing diol in quenching the polymerization of a difunctional epoxy formulation. Differently from the monosulfide, the propagation can start, and only at high sulfur:epoxy ratio the reactions of the disulfides with the oxonium ions effectively compete with the propagation and stops the polymerization. This inhibition mechanism can be exploited as a novel maskless photolithographic approach, enabling the spatially controlled patterning of epoxy coatings through localized deposition of the disulfide diol. As a proof of concept, sharply defined features with sizes down to 200 µm are successfully fabricated. These results introduce disulfide‐mediated polymerization quenching as a versatile and material‐efficient method for epoxy photopatterning.

## Graphical abstract

This study investigates the role of disulfide‐containing molecules on the photopolymerization of epoxy monomers through cationic ring‐opening reaction, with the demonstration of the selective inhibition of the polymerization when disulfides are added to the photocurable formulation. This quenching is then exploited as a novel maskless photolithographic technique to pattern epoxy coatings with features as small as 200 µm.

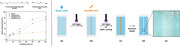

## 1 Introduction

Over the past decades, photoinduced ring‐opening polymerization (ROP) of epoxy monomers was affirmed as a well‐established polymerization technique, mainly due to its wide range of applications, e.g., coatings, adhesives, microelectronics, or 3D printing, both at laboratory and industrial scale [1, 2, 3, 4]. Epoxide cationic photopolymerization offers several advantages compared to the free‐radical mechanism, chief among them being the lack of oxygen inhibition, as well as good adhesion to various substrates, higher mechanical properties, lower shrinkage, and enhanced chemical resistance [5, 6, 7, 8, 9]. Furthermore, the ability to “dark cure” and proceed even when light is switched off, due to the long‐living cationic propagating species, makes this process particularly attractive for the polymerization of thick samples or colored and filled systems [7, 10, 11, 12, 13].

The cationic polymerization of epoxy monomers is initiated with the protonation of epoxy groups by strong Brønsted acids, generated upon photocleavage of onium salts (e.g., triarylsulfonium or diaryliodonium) [14, 15]. The propagation is then driven by a chain growth mechanism, which proceeds with the addition of epoxy monomers to the protonated oxonium ions through a ring‐opening reaction [16]. When cationic photopolymerization is carried out in the presence of alcohols, the ring‐opening proceeds with the so‐called *activated monomer mechanism*. This mechanism involves the transfer of protons from the alcohol to a new epoxy monomer, thereby initiating a new propagating chain [1, 5, 17, 18, 19, 20]. The presence of the nucleophilic hydroxy group thus leads to a deep change in the reaction process and in the final properties of the material [6, 18, 21, 22]. Moreover, the presence of amines has been extensively studied as a possibility to improve the control of the cationic polymerization mechanism, avoiding side reactions, and regulating byproduct formation [23, 24]. Besides alcohols and amines, several studies have investigated the role of sulfides and thiols in the curing of epoxide and vinyl ethers [23, 25, 26]. In these cases, the lone pair of sulfur atoms promptly reacts with the carbonium ions [23, 25] and the polymerization is inhibited [25] due to the high stability of the obtained sulfonium ion, whose formation is then favored compared to the propagation of epoxide. Inhibition of a photoinduced reaction can serve as a principle to create specific spatial arrangements or surface patterns. To generate defined patterns, traditional lithographic methods that rely on predesigned masks are now overcome by maskless photolithography [27, 28, 29, 30] which allows direct projection or deposition of the desired pattern onto the photoresist [27]. Such method offers several advantages, including higher scalability, reduced contamination risk, and lower fabrication costs associated with mask production [23, 28, 29, 30]

Herein, we investigate the influence of a disulfide compound bearing hydroxy groups on the cationic photopolymerization of epoxides. Conditions enabling controlled inhibition of the polymerization process are identified and subsequently exploited to demonstrate a novel maskless photolithographic strategy for fabricating well‐defined epoxy patterns without the use of physical masks.

## 2 Results and Discussion

### 2.1 Disulfide Effect on Epoxide Photopolymerization

**FIGURE 1:**
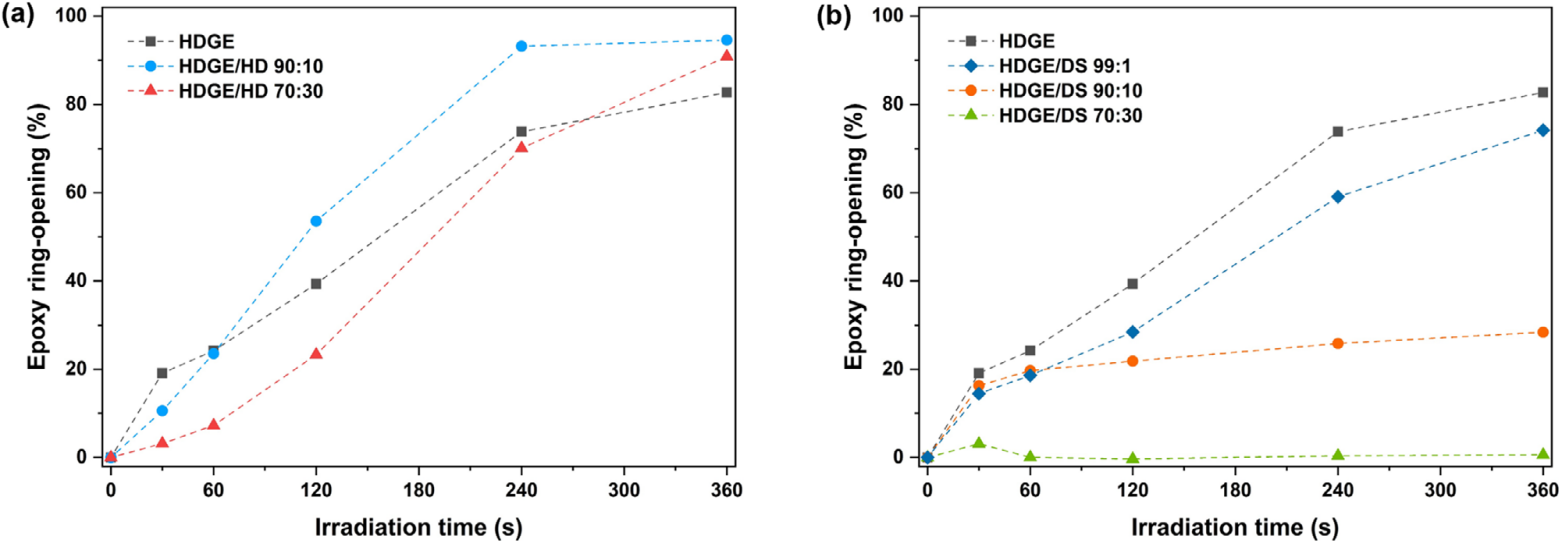
Ring‐opening of epoxy groups over irradiation time for (a) HDGE/HD and (b) HDGE/DS systems.

**SCHEME 1:**
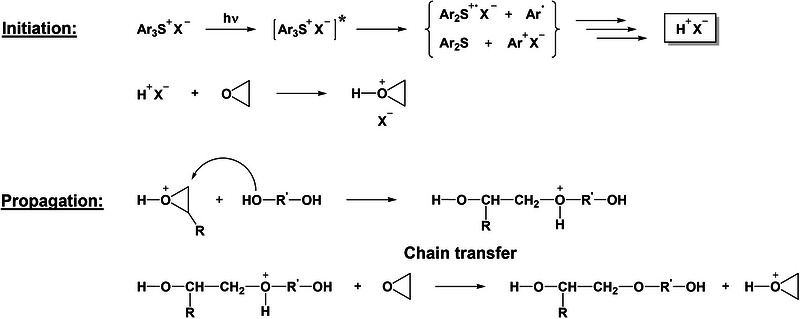
Cationic ring‐opening polymerization reaction in the presence of a diol as CTA.

In this study, we investigated the cationic photopolymerization of 1,6‐hexanediol diglycidyl ether (HDGE) in the presence of two diols with comparable chain lengths: 1,6‐hexanediol (HD), which contains a six‐carbon aliphatic chain, and 2‐hydroxyethyldisulfide (DS), which has a four‐carbon backbone and a disulfide linkage. The extent of epoxide ring opening as a function of irradiation time was monitored using Fourier Transform Infrared (FTIR) spectroscopy (Figure 1), and the gel content was also determined. The epoxide conversion was estimated through the change of absorbance of the C‐O stretching band centered at 910 cm^−1^ (see Figures S1 and S2). In accordance with the existing literature [5, 7, 12], HD acted as a chain transfer agent (CTA), as illustrated in Scheme 1. Its presence significantly influenced the polymerization kinetics, causing a pronounced reduction in both the conversion and the polymerization rate during the first minute of irradiation. At longer irradiation times, however, HD promoted a higher final conversion (nearly 95%) compared to the neat epoxy monomer HDGE, which reached approximately 80% conversion after 360 s of irradiation (Figure 1a).

A comparison of the conversion curves further showed that both the initial polymerization rate R_p,i_ and the propagation rate R_p,p_ (Table 1) depend on the hydroxy:epoxy ratio. When this ratio was 0.1 (HDGE/HD 90:10), the propagation rate increased, as reported in the literature, despite the initial lowering of R_p,i_. The same trend was observed at a ratio of 0.4 (HDGE/HD 70:30), although in this case a dilution effect might arise, partially offsetting the rate‐enhancing contribution of the hydroxy groups [21, 31, 32, 33, 34]

**TABLE 1:** Gel fraction values, initial polymerization rate R_p,i_ (t = 0–10 s) and propagation rate R_p,p_ (t = 60–240 s) for HDGE, HDGE/HD and HDGE/DS samples.

Sample	Gel fraction [%]	R_p,i_ [s^−1^]	R_p,p_ [s^−1^]
HDGE	95.0 ± 0.3	6.5 × 10^−3^	2.7 × 10^−3^
HDGE/HD 90:10	93.2 ± 0.2	3.5 × 10^−3^	3.9 × 10^−3^
HDGE/HD 70:30	83.8 ± 0.4	1.1 × 10^−3^	3.5 × 10^−3^
HDGE/DS 99:1	94.9 ± 0.3	5.9 × 10^−3^	2.3 × 10^−3^
HDGE/DS 90:10	Fully soluble	5.4 × 10^−3^	3.4 × 10^−4^
HDGE/DS 70:30	Fully soluble	0.7 × 10^−3^	2.1 × 10^−5^

In the presence of HD as CTA, the gel fractions of the samples (Table 1) decreased only slightly. This behavior arose from the bifunctionality of HD. When a difunctional diol reacts with the growing polymer chain, it produces a hydroxy‐terminated polymer and releases a free proton capable of initiating further monomer activation. Therefore, the overall polymerization is not terminated, as would occur with monofunctional CTAs [31], and the network density is largely preserved.

A remarkably different trend was noted when DS was added to HDGE. As shown in Figure 1b, when the DS diol content was not exceeding 1 mol% (sulfur:epoxy ratio 0.01), the polymerization proceeded with a negligible reduction of the epoxy conversion. When the DS amount increased, the extent of epoxide ring‐opening dropped: the value of the final conversion was as low as 28% for a sulfur:epoxy ratio of 0.1 (HDGE/DS 90:10), while a near complete inhibition occurred when the ratio was 0.4 (HDGE/DS 70:30). Accordingly, in the FTIR spectra, shoulders around 3000 cm^−1^, corresponding to C‐H bonds present in the unreacted epoxy monomer, were evident confirming that the polymerization did not take place (see Figure S2). In both cases, the resulting polymers remain very tacky and completely soluble in dichloromethane, indicating the absence of any gel formation (Table 1).

Excluding the system with the highest DS content, which experienced complete inhibition, the initial polymerization rate R_p,i_ was only slightly reduced by the presence of the disulfide CTA (Table 1). Whereas the propagation rate R_p,p_ was strongly affected by the amount of disulfide: for the HDGE/DS 70:30 formulation, R_p,p_ was approximately one order of magnitude lower than that of neat HDGE.

**SCHEME 2:**
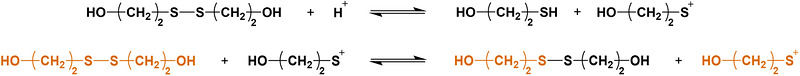
Proton‐induced disulfide cleavage of DS.

Overall, in contrast to HD, the addition of DS to the monomer did not induce any reduction in the initial polymerization rate, nor did it lead to an increase in conversion or an enhancement of the polymerization rate. The different effect of the two diols on the photopolymerization of HDGE could clearly be ascribed to the presence of the disulfide bond in DS. In fact, many reactions involving sulfur could play a role in the system. As reported in previous studies [35, 36], disulfides are susceptible to attack by electrophile species, e.g., protons, which can interact with the 3p electrons of the sulfur atoms. This interaction leads to the protonation of the S‐S bond and its subsequent cleavage, with the formation of a thiol and a sulfonium ion, which gives an exchange reaction as illustrated in Scheme 2 [35, 36]. Consistent with this, acid‐initiated cationic ROP of cyclic disulfide monomers (i.e., 1,2‐dithiolanes) has also been recently reported in the literature [35], with the sulfonium ion acting as a key intermediate enabling chain propagation through nucleophilic attack. Sulfonium ions and disulfide groups can then undergo multiple exchange reactions where the S‐S bonds are continuously broken and reformed.

If the disulfide‐related reactions described above occurred, no epoxy conversion would be expected, even in the presence of the lowest DS concentration. An estimation of the molar amount of H^+^ generated by the photoinitiator (see Table S1) indicated that the proton concentration was substantially lower than the number of available disulfide bonds in any system under investigation. Moreover, as suggested by the equilibria shown in Scheme 2, if protons were indeed being consumed through interaction with disulfide groups, formation of thiols and sulfonium ions could be expected. In contrast, FTIR analysis did not reveal any absorption band near 2550 cm^−^^1^, characteristic of the S‐H stretching vibration of thiols (see Figure S2).

**SCHEME 3:**
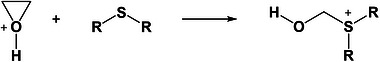
Oxonium ion‐sulfonium ion equilibrium.

A reaction path previously established for the cationic photopolymerization of epoxides in the presence of dimethylsulfides is sketched in Scheme 3. Having remarkable nucleophilicity, the monosulfide displays typical inhibiting characteristics, promoting the formation of sulfonium ions after the initial protonation of the epoxide. The equilibrium between the oxonium and the sulfonium ion always lies in favor of the latter species, given its lower basicity (pK_b_ ≈ 7 for a typical epoxide, while ≈ 11 for a sulfide such as dimethylsulfide). As sulfonium ions are relatively stable, Scheme 3 represents a termination mechanism, commonly referred to as ion trapping, which stops the propagation [23, 26]. Crivello et al. reported that even 1 mol% of a sulfide is enough to have complete inhibition of epoxide polymerization. However, when the DS disulfide was used at the same concentration, no inhibition of the photopolymerization was observed; instead, only a slight decrease in epoxide conversion was detected upon irradiation (Figure 1b). This evidence suggests that the ROP propagation may occur, while full inhibition takes place only strongly increasing the DS amount.

**SCHEME 4:**
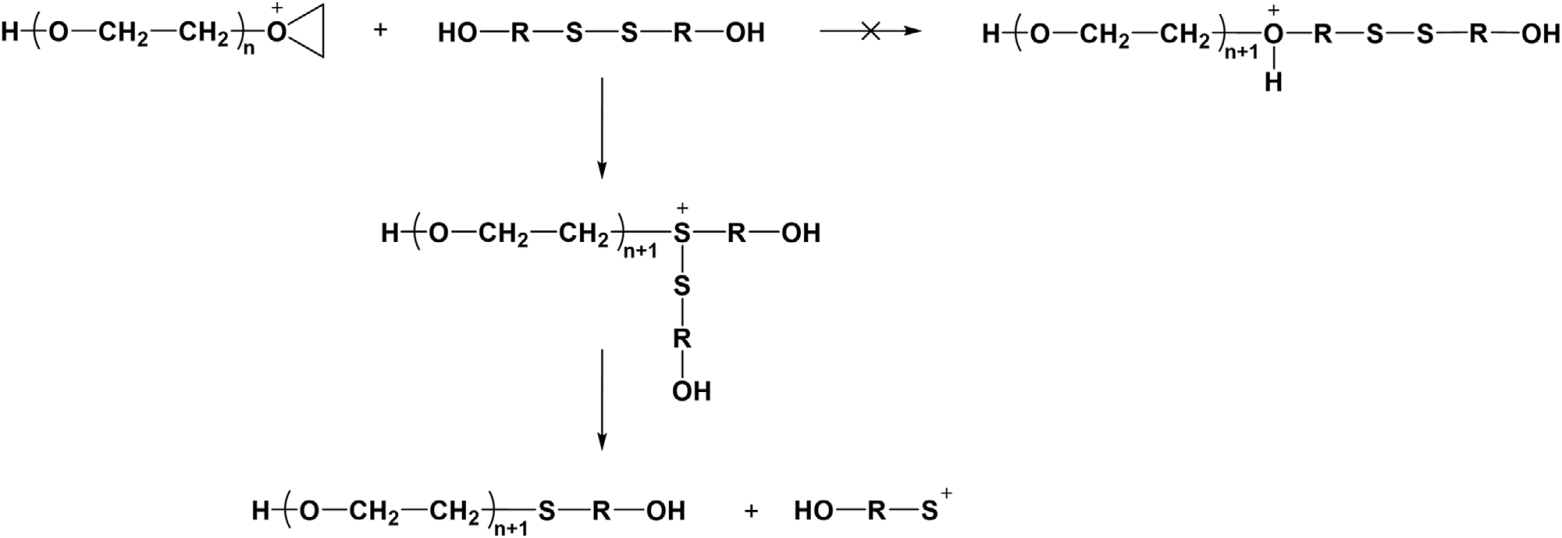
Proposed reaction mechanism between propagating oxonium ions and disulfide‐containing diols.

Considering the reactions depicted in Scheme 2 and Scheme 3, the proposed reaction pathway for the system under investigation is sketched in Scheme 4. Upon UV activation, initiation proceeds normally, and growing chains bearing oxonium end groups are formed. These oxonium species enter into equilibrium with the disulfide, which competes with both chain propagation and chain‐transfer processes. The equilibrium generates a sulfonium ion, which subsequently undergoes thiolysis; this step yields a thioether and a new sulfonium species, enabling a self‐propagating sequence of side reactions. In short, the disulfide effectively competes with chain propagation and chain transfer processes: macrosulfonium ions are formed, quenching the growing chain, and are subsequently converted into short ionic fragments (HO‐R‐S^+^) together with hydroxy‐monosulfide products. These species can continue to react with oxonium groups, thereby further terminating propagating chains and reducing overall polymerization efficiency.

Furthermore, as reported in the literature [36], the sulfonium ions generated in this system could attack additional S‐S bonds, leading to a series of disulfide‐exchange cascade reactions within the formulation. The low nucleophilicity of the non‐coordinating hexafluorophosphate anion (PF_6_^−^) from the triaryl sulfonium salt photoinitiator keeps the newly‐formed sulfonium ions in an active electrophilic state, thus supporting the propagation of the disulfide exchange reaction [35, 37]. Such exchange and side reactions can occur and are likely to compete with the epoxide photopolymerization.

**SCHEME 5:**
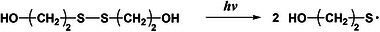
Light‐induced disulfide homolysis.

Another reaction pathway that may compete with cationic epoxide photopolymerization is the homolytic cleavage of the S‐S bond, generating thiyl radicals: disulfide bonds are photolabile under UV irradiation, and can compete with the photoinitiator for photon absorption during irradiation (Scheme 5), thereby introducing additional radical processes into the system.

To better investigate the effect of DS, mixtures containing only HDGE and the photoinitiator, excluding the diols, were first irradiated, and subsequently the diols were added after the reaction had been initiated. This approach was designed to minimize both the proton‐induced disulfide cleavage (Scheme 2) and the light‐triggered homolytic S‐S bond scission (Scheme 5), which could both compete with cationic polymerization. In detail, HDGE and triaryl sulfonium salts photoinitiator were mixed into a beaker and exposed to UV light for 20 s to trigger the photoacid generation and, thus, the protonation of the epoxy rings. An irradiation time of 20 s was selected because it was sufficient to form the strong acid required to initiate cationic polymerization, while still resulting in only around 3.5% epoxide ring‐opening. At this conversion, the mixture remained sufficiently low in viscosity to allow efficient incorporation and mixing of the diols.

**FIGURE 2:**
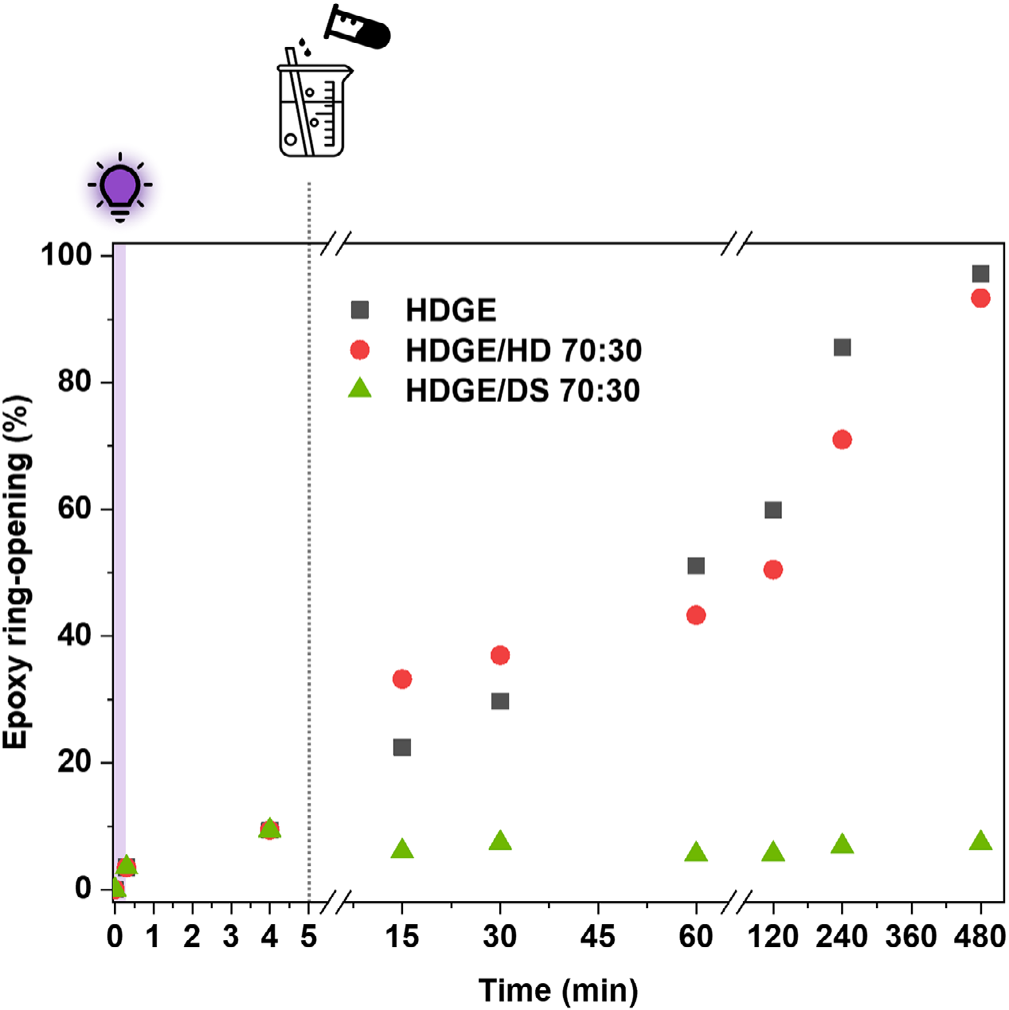
Ring‐opening of epoxy groups over time in dark curing. Irradiation of samples was carried out in the absence of diol for 20 s with a UV light intensity of 100 mW cm^−2^. Corresponding diol amounts (30 mol%) were introduced after 5 min.

The diol (30 mol%) was added to the pre‐irradiated mixture 5 min after the irradiation was stopped. Following brief stirring, the formulations were poured into a glass petri dish and stored in a dark environment to allow dark curing (i.e., continued polymerization that proceeds after the light source is switched off, driven by long‐lived reactive species). For comparison, the same procedure was applied to HDGE without diols to provide a reference system. The evolution of the epoxy ring‐opening extent during dark curing is shown in Figure 2. In the 8 h following diol addition, both HDGE and HDGE/HD 70:30 formulations continued to polymerize in the dark, reaching final conversions of 97% and 93%, respectively. As expected, the cationic ROP proceeded even without continuous irradiation, reaching quantitative conversions, higher than those reached at the end of a longer irradiation time (360 s) and yielding solid crosslinked samples with similar gel content to the previous ones (Table 2). In contrast, when DS was added, the epoxy ring‐opening reaction completely stopped: the conversion remained fixed at the value attained prior to diol addition, and the resulting material remained completely soluble in dichloromethane (Table 2), indicating complete inhibition of crosslinking.

**TABLE 2:** Gel fraction values of HDGE, HDGE/HD and HDGE/DS samples cured in dark conditions. Irradiation of samples was carried out in the absence of the diol for 20 s with a UV light intensity of 100 mW cm^−2^. The diol was introduced after 5 min, and the gel content was evaluated after 8 h of samples storage in the dark.

Sample	Gel fraction [%]
HDGE	97.7 ± 0.4
HDGE/HD 70:30	83.8 ± 0.2
HDGE/DS 70:30	Fully soluble

**FIGURE 3:**
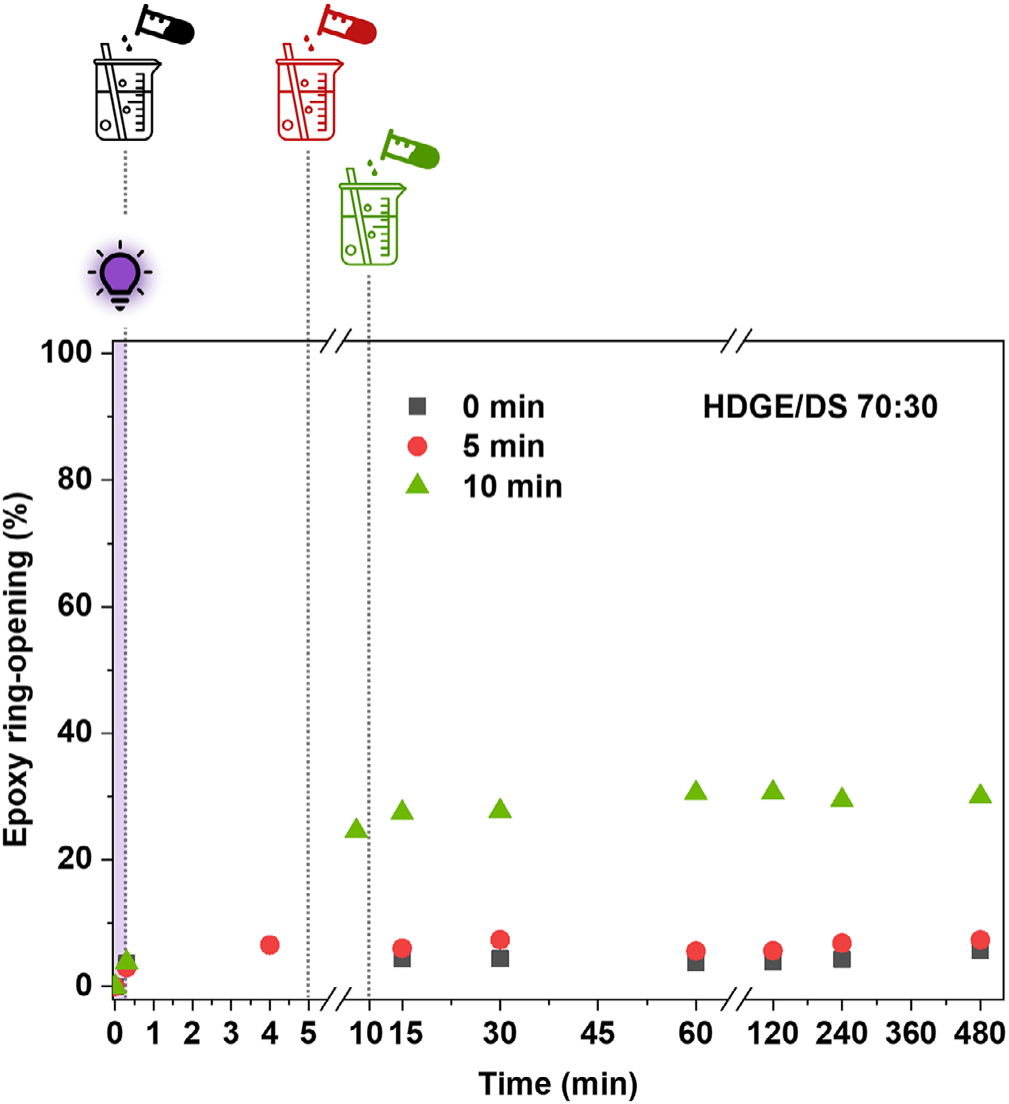
Ring‐opening of epoxy groups over time in dark curing with different waiting times (between irradiation and DS addition) for HDGE/DS 70:30. Irradiation of samples was carried out for 20 s with a UV light intensity of 100 mW cm^−2^. Corresponding diol amounts (30 mol%) were introduced after 0, 5, and 10 min.

Increasing the time between the end of the irradiation and the DS addition confirmed this behavior: polymerization stopped as soon as DS was added to the formulation, as shown in Figure 3. Regardless of whether the diol was introduced 0, 5, or 10 min after irradiation, the epoxy ring‐opening conversion remained constant, indicating that once DS is added, the propagation process is irreversibly halted. This time‐independent inhibition suggests that the disulfide does not interfere with photoacid generation or initial protonation of the epoxy groups but rather interacts with the growing chains, effectively deactivating them and preventing further polymer growth.

### 2.2 Exploiting Disulfide Diols Polymerization Inhibition for Patterning

It has been shown that the epoxy cationic ROP can be either completely inhibited when a sufficient amount of disulfide bonds is present during irradiation or selectively stopped on demand upon addition of a disulfide after propagation has already started. This time‐controlled inhibition could be turned into a photopolymerization spatial control, enabling the generation of defined patterns within epoxy coatings.

**FIGURE 4:**
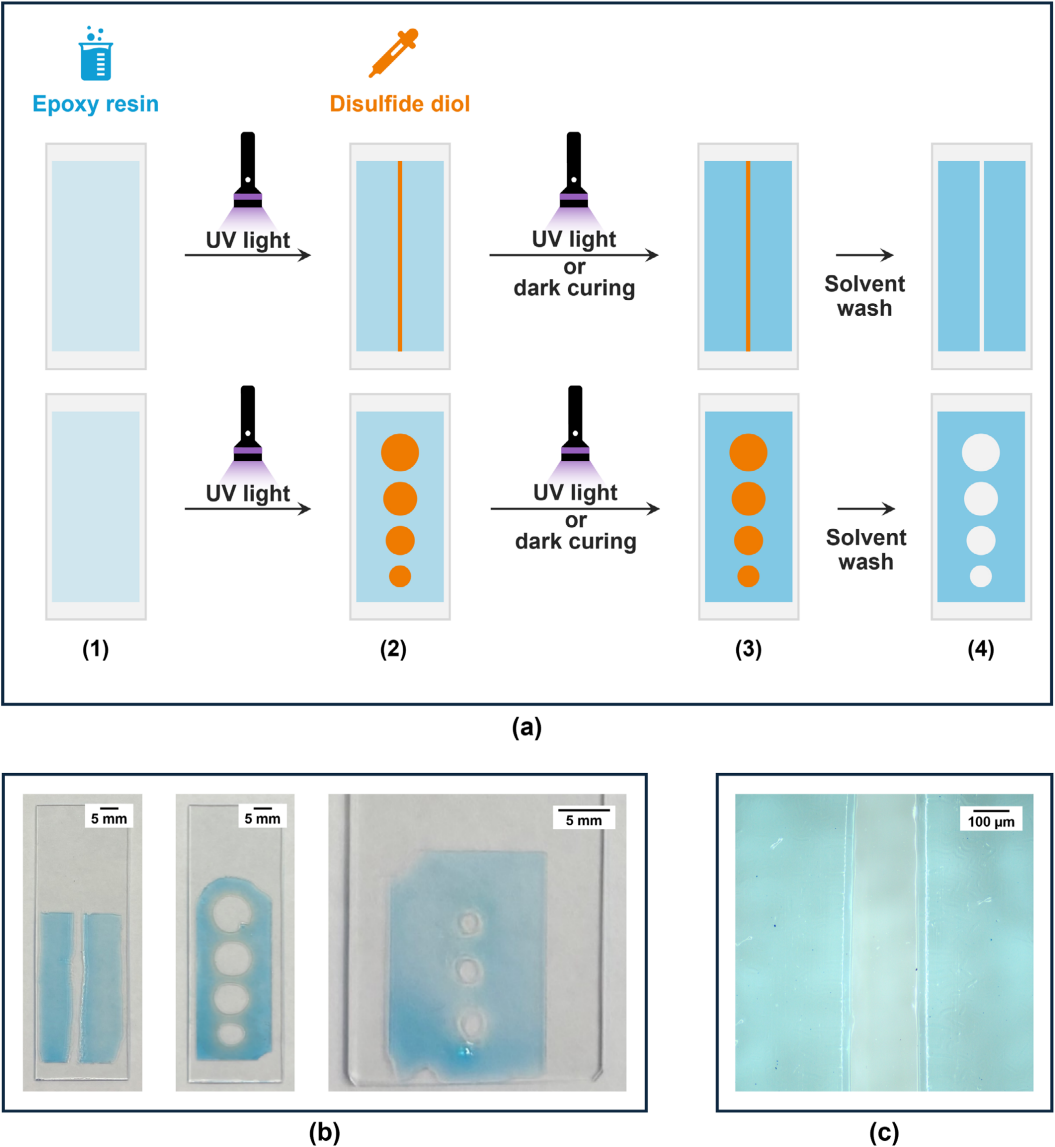
(a) Schematic representation of the maskless disulfide‐mediated cationic photolithography method to pattern epoxy coatings: (1) deposition of the photocurable epoxy resin optional initial UV exposure, (2) localized deposition of DS in specific areas to define the pattern, (3) UV curing of epoxy coating, with polymerization inhibited in DS‐containing regions, (4) solvent development to remove uncrosslinked material and reveal the patterned structures. Examples of resulting patterns obtained on (b) HDGE and (c) 3,4‐epoxycyclohexylmethyl 3,4‐epoxycyclohexanecarboxylate coatings. The epoxy resins were premixed with blue dye to enhance pattern visibility.

A scheme of the maskless photolithographic process exploiting this inhibition mechanism is shown in Figure 4a. First, the neat photocurable epoxy monomer containing the photoinitiator was applied on a glass substrate to form a thin film (step 1). The film was then irradiated for a predefined duration, selected according to the targeted conversion, and a disulfide‐based formulation was subsequently dispensed onto the film to define the pattern (step 2). Crosslinking of the epoxy network was obtained either through a secondary UV exposure (faster) or by dark curing (slower), while in the areas where the disulfide diol was present, the epoxide polymerization was locally inhibited (step 3). A final development step was carried out to obtain the expected structures (step 4). In the areas containing the disulfide additive, the epoxy coating remained soluble in the development solvent and was selectively removed, yielding well‐defined features. It should be noted that the initial irradiation step may be omitted; the process parameters must be adjusted according to formulation viscosity, desired pattern geometry, and the required degree of conversion.

This patterning approach was successfully demonstrated not only with HDGE but also with other epoxides, including 3,4‐epoxycyclohexylmethyl 3,4‐epoxycyclohexanecarboxylate. Epoxy films with a thickness of 50 µm were patterned using a DS‐based formulation containing a thickening agent to prevent uncontrolled spreading. The formulation was applied by syringe to create either continuous lines or discrete droplets. Representative patterns obtained on the two epoxy systems are shown in Figure 4b,c. The resulting features exhibited sharply defined features with lateral dimensions as small as 200 µm, achieved without the need for optical masks or physical templates/molds. These results served as proof of concept for maskless disulfide‐mediated cationic epoxy photolithography, showcasing a novel strategy for spatially controlled photocross‐linking based on selective inhibition. It is important to consider that the deposition method, as well as the solubility in the resin and the viscosity of the inhibitory formulation, are critical parameters governing pattern quality, precision, and resolution. Precise deposition ensures accurate spatial confinement of the inhibition zones, directly affecting the fidelity of the patterned features. Adequate solubility and viscosity minimize diffusion and spreading into the surrounding resin, preserving feature sharpness. In this context, the use of DS instead of conventional disulfides is advantageous, as the terminal ‐OH groups enable versatile functionalization to finely tune the inhibitor properties.

## 3 Conclusions

In this study, disulfide inhibition of epoxy cationic ring‐opening photopolymerization was investigated and demonstrated. Results support a mechanism in which disulfides do not intervene in the initiation events but deactivate the growing chains, suppressing network formation. Interestingly, the inhibition can be triggered on demand by adding the disulfide after irradiation, enabling temporal control over the polymerization. This controllable inhibition was further exploited to propose a maskless, disulfide‐mediated photolithographic technique, enabling spatial patterning of epoxy coatings without the use of optical masks or molds. As a proof of concept, sharply defined structures with feature sizes down to 200 µm were obtained through localized deposition of the disulfide diol onto a photocurable epoxy layer.

## 4 Experimental Section

### 4.1 Materials

**FIGURE 5:**
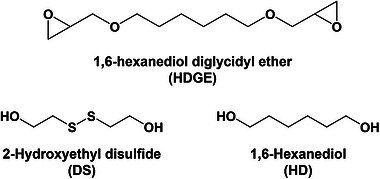
Chemical structures of the difunctional epoxy monomers and difunctional alcohols used in this study.

1,6‐hexanediol diglycidyl ether (HDGE) was kindly supplied by Huntsman Corporation. 1,6‐hexanediol (HD), 2‐hydroxyethyl disulfide (DS), and polyethylene glycol (M_w_ = 1 000 000 g mol^−1^) (PEO) were purchased from Sigma–Aldrich. Triarylsulfonium hexafluorophosphate salt (as a 50 wt.% solution in propylene carbonate) was also purchased from Sigma–Aldrich and used as a photoinitiator for cationic polymerization. Blue and orange pigments were gently provided by Rahn AG. The chemical structures of the epoxy monomers and diols used in this research work are reported in Figure 5.

All the listed reagents were used as received, without any further purification. All other chemicals used that were not listed above were purchased from Sigma‐Aldrich.

### 4.2 Preparation of Epoxy/Diol Formulations

The photocurable formulations were prepared by adding different molar amounts of the two diols (HD or DS) to the epoxy monomer HDGE. The sulfonium salt photoinitiator was added at 2 wt.% with respect to the epoxide/alcohol mixtures. The composition of all the formulations tested is reported in Table 3.

**TABLE 3:** Composition of photocurable formulations.

Formulation	HDGE [mol%]	HD [mol%]	DS [mol%]	Photoinitiator [wt%]
HDGE	100	—	—	2
HDGE/HD 90:10	90	10	—	2
HDGE/HD 70:30	70	30	—	2
HDGE/DS 99:1	99	—	1	2
HDGE/DS 90:10	90	—	10	2
HDGE/DS 70:30	70	—	30	2

Formulations were prepared and mechanically stirred for 15 min to ensure the complete dispersion of the photoinitiator. While all samples were prepared at room temperature, the formulations containing HD were stirred at 50°C for 10 min before adding the photoinitiator to melt HD crystals, allowing a better homogenization of the formulations.

For the patterning experiments, a DS/PEO formulation was prepared by mixing DS with 2.5 wt.% PEO overnight to allow complete dispersion and homogenization.

### 4.3 Photocuring of Epoxides

The UV‐curable formulations were coated onto a glass substrate using a wire‐wound applicator with variable pitches. All the formulations were cured using a medium‐pressure Hg lamp (Dymax ECE 5000 Flood Lamp, Dymax) using an intensity of 100 mW cm^−2^. The UV light intensity was assessed through a UV Power Puk II (EIT Instrument Markets). After irradiation, the samples were stored in the dark to allow dark curing to proceed. According to a well‐established methodology [12], 24 h after photocuring, epoxide samples were placed in a closed system saturated with 5 vol% water/ammonia solution vapors for 15 min to quench any remaining acidic species.

Selected epoxide/diol formulations (HDGE/HD 70:30 and HDGE/DS 70:30) were also cured following a different protocol. 5 g of HDGE mixtures with 2 wt.% cationic photoinitiator but without diols were placed in a 20 mL beaker and irradiated for 20 s using a medium‐pressure Hg lamp (Dymax ECE 5000 Flood Lamp, Dymax) with an intensity of 100 mW cm^−2^. The corresponding diol amount (30 mol%) was then added to the activated mixture at different intervals and dispersed homogeneously by briefly stirring the formulation. HDGE/diol formulations were subsequently poured into a glass petri dish and left in a dark environment overnight to allow complete curing. A neat epoxy formulation without any diol was also prepared, cured with the same procedure, and used as a reference.

### 4.4 Characterization

For evaluating the conversion of the reactive groups during the photopolymerization reaction, FTIR spectroscopy analyses were performed using a Thermo Fisher Scientific Nicolet is50 spectrometer. HDGE films with a thickness of around 10 µm were spread on a silicon wafer through a wire‐wound applicator, irradiated with specific UV light, and analyzed by FTIR spectroscopy in transmission mode in the spectral range of 4000–400 cm^−1^, collecting 32 scans per spectrum with a resolution of 4 cm^−1^. The conversion of samples was also investigated in Attenuated Total Reflectance mode (ATR) at defined time intervals using the same instrument. The extent of the epoxy ring‐opening conversion was calculated using the following equation (Equation 1):

(1) $$\mathrm{Conversion}\;\left(\%\right)=\left(1-\frac{A_{t}}{A_{0}}\right)\;\times 100$$

in which $A_{t}$ is the area of the characteristic peak of the epoxy group (C‐O) at time t, and $A_{0}$ is the area of the same peak at time 0. Peaks were selected depending on the chemical structure of the analyzed epoxy monomer. C‐O stretching peaks centered at 910 cm^−1^ were used, and peak areas were normalized using the C‐H stretching peak at 2860 cm^−1^ as a reference. The first derivative of the epoxy ring opening conversion was calculated to evaluate the photopolymerization rate of the reactive mixtures R_p_, according to the following equation (Equation 2):

(2) $$R_{p}=\frac{d\left(conversion\right)}{dt}\;$$

The initial polymerization rate R_p,i_ was determined over the first 10 s of the reaction, whereas the propagation rate R_p,p_ was evaluated in the 60–240 s irradiation interval.

Gel content analyses assessed the insoluble fraction of UV‐cured samples by measuring weight loss after 24 h of extraction in dichloromethane at room temperature.

### 4.5 Patterning

Patterned samples were made as follows: first the neat epoxy formulation was coated on a glass slide to form a liquid film around 50 µm thick. With the aid of a syringe pump equipped with a 27‐gauge needle (SKE Research Equipment), DS in the presence of a thickener (a high molecular weight polyethylene glycol 2.5 wt.%) was deposited on top of the epoxy films in the form of a continuous line or as drops. The systems were then cured for 240 s under UV light using a medium‐pressure Hg lamp (Dymax ECE 5000 Flood Lamp, Dymax) with an intensity of 100 mW cm^−2^. Once cured, samples were rinsed with ethanol to remove the uncured formulation and dried at room temperature to allow complete solvent evaporation. To enhance the visibility of the patterns, a small amount of dye was added to the formulations.

## Funding

This work was supported by the Italian Ministry of University and Research (MUR) [DM 1061/2021 PON‐Dottorati di ricerca su tematiche green e dell'innovazione].

## Conflicts of Interest

The authors declare no conflict of interest.

## Supporting information




**Supporting File**: marc70229‐sup‐0001‐SuppMat.docx.

## Data Availability

The data that support the findings of this study are available from the corresponding author upon reasonable request.
