# Supplementary material for: Disulfide‐Induced Inhibition of Epoxy Cationic Photopolymerization: A Route to Maskless Patterning
Source: Macromol Rapid Commun. 2026 Feb 5;47(13):e00956. doi: 10.1002/marc.202500956 (PMC13331524; doi:10.1002/marc.202500956)
Supplement: Supplementary file 1 — Supporting File: marc70229‐sup‐0001‐SuppMat.docx. [file MARC-47-e00956-s001.docx]

Supporting Information

Disulfide-induced inhibition of epoxy cationic photopolymerization: a route to maskless patterning

Alberto Spessa*, Roberta Bongiovanni, and Alessandra Vitale*

**Table S1.** Molar amount of epoxy groups, disulfide bonds, and protons present in HDGE/DS formulations. All the molar amounts were calculated considering a single mole of formulation and 2 wt% of cationic photoinitiator, together with the production of 1 moles of H^+^ for each mole of photoinitiator present (based on the reaction scheme proposed in^[16]^) .

| Formulations | Epoxy groups [mol] | Disulfide bonds [mol] | H^+^ [mol] |
| --- | --- | --- | --- |
| HDGE | 2 | - | 0.0017 |
| HDGE/DS 99:1 | 1.98 | 0.01 | 0.0017 |
| HDGE/DS 90:10 | 1.8 | 0.1 | 0.0017 |
| HDGE/DS 70:30 | 1.4 | 0.3 | 0.0017 |


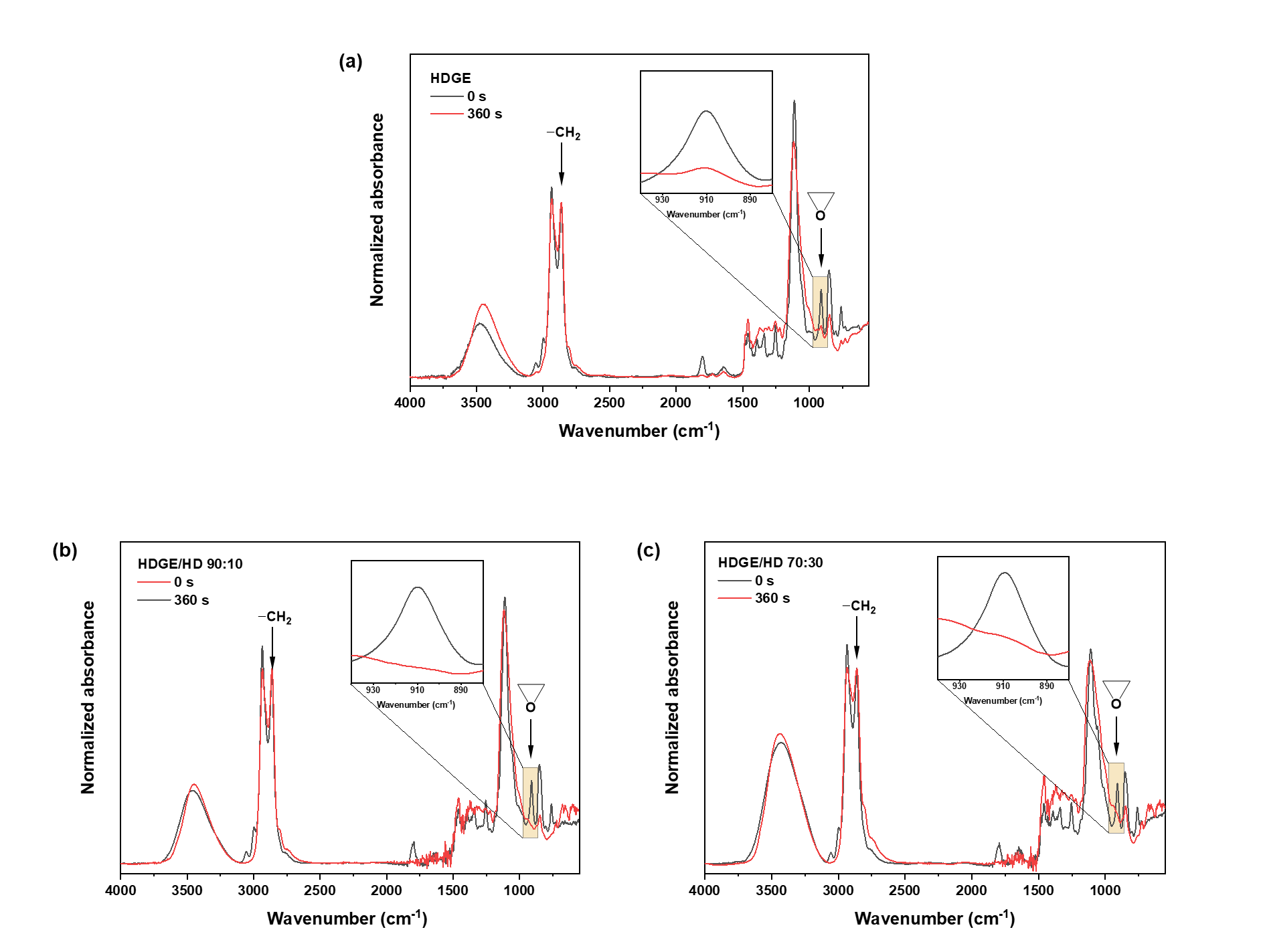


**Figure S1.** FTIR spectra of HDGE (a), HDGE/HD (b) 90:10 and (c) 70:30 formulations before and after 360 s of irradiation. Spectra were normalized with the intensity of the CH stretching peak at 2860 cm^-1^.


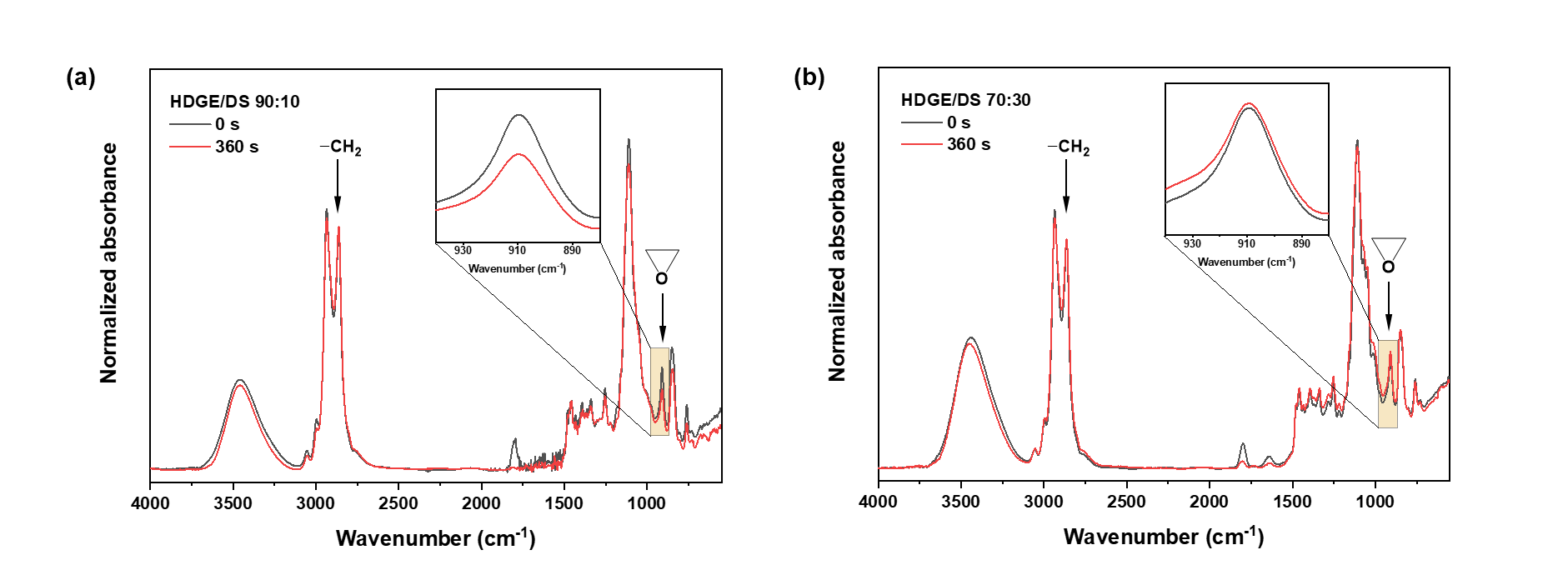


**Figure S2.** FTIR spectra of HDGE/DS (a) 90:10 and (b) 70:30 formulations before and after 360 s of irradiation. Spectra were normalized with the intensity of the CH stretching peak at 2860 cm^-1^.
